# High-fructose feeding does not induce steatosis or non-alcoholic fatty liver disease in pigs

**DOI:** 10.1038/s41598-021-82208-1

**Published:** 2021-02-02

**Authors:** Nikolaj H. Schmidt, Pia Svendsen, Julián Albarrán-Juárez, Søren K. Moestrup, Jacob Fog Bentzon

**Affiliations:** 1 Department of Clinical Medicine, Heart Diseases, Aarhus University, Aarhus, Denmark; 2 Institute of Molecular Medicine, University of Southern Denmark, Odense, Denmark; 3 Department of Biomedicine, Aarhus University, Aarhus, Denmark; 4 Department of Clinical Biochemistry and Pharmacology, Odense University Hospital, Odense, Denmark; 5 Centro Nacional de Investigaciones Cardiovasculares (CNIC), Madrid, Spain; 6 Steno Diabetes Center Aarhus, Aarhus, Denmark

**Keywords:** Non-alcoholic fatty liver disease, Experimental models of disease, Genetics research, Acute inflammation

## Abstract

Non-alcoholic fatty liver disease (NAFLD) is an increasingly prevalent condition that has been linked to high-fructose corn syrup consumption with induction of hepatic de novo lipogenesis (DNL) as the suggested central mechanism. Feeding diets very high in fructose (> 60%) rapidly induce several features of NAFLD in rodents, but similar diets have not yet been applied in larger animals, such as pigs. With the aim to develop a large animal NAFLD model, we analysed the effects of feeding a high-fructose (HF, 60% w/w) diet for four weeks to castrated male Danish Landrace-York-Duroc pigs. HF feeding upregulated expression of hepatic DNL proteins, but levels were low compared with adipose tissue. No steatosis or hepatocellular ballooning was seen on histopathological examination, and plasma levels of transaminases were similar between groups. Inflammatory infiltrates and the amount of connective tissue was slightly elevated in liver sections from fructose-fed pigs, which was corroborated by up-regulation of macrophage marker expression in liver homogenates. Supported by RNA-profiling, quantitative protein analysis, histopathological examination, and biochemistry, our data suggest that pigs, contrary to rodents and humans, are protected against fructose-induced steatosis by relying on adipose tissue rather than liver for DNL.

## Introduction

Non-alcoholic fatty liver disease (NAFLD) is closely related to lifestyle and estimated to be the most prevalent liver disease in the world affecting ~ 75–100 million people in USA alone^1^. The pathogenesis behind NAFLD is diverse and not fully understood, but genetic predisposition, the intestinal microbiome, insulin resistance, and the intake of fat and fructose may play a role in a “multiple hit” model^2^. Ultimately, fat accumulation in the liver (NAFLD) triggers an inflammatory response, i.e. non-alcoholic steatohepatitis (NASH), that secondarily may progress to development of fibrosis and cirrhosis.

Fructose consumption has long been speculated to play a role in the development of NAFLD and its progression to NASH^3^. Fructose up-regulates key enzymes for hepatic de novo lipogenesis (DNL), probably driven by extensive first-pass metabolism after intestinal absorption, and increased fructose consumption has been correlated with progression of fibrosis in a NAFLD population^4–6^. The link between high-fructose consumption and the development of NAFLD/NASH histological changes has been particularly clear in rodents, and feeding rats and mice very high-fructose diets (> 60%) is currently among the most widely used experimental models. High fructose-feeding in these models leads to 30–800% increase in hepatic fat accumulation; macrovesicular steatosis in 5% and up to 66% of lobules; and lobular inflammation with 2–4 foci per 200 × field at microscopic examination^7–10^.

The development of similar models in large animals for experimental studies would facilitate research with a direct translational outlook, allowing repeated tissue sampling with liver biopsies and the use and development of clinical imaging techniques. Efforts have already been made to establish porcine models showing histopathological steatosis progressing to portal and lobular inflammation, hepatocyte ballooning and fibrosis. Lee et al. fed male/female Ossabaw pigs (from 5 to 10 months of age) various diets for 24 weeks, and found no steatosis, ballooning or fibrosis when pigs received diets supplemented with moderate amounts of fructose (20% calories) and fat (10.5% calories)^11^. In contrast, Liang et al. observed both hepatocyte ballooning and fibrosis but no macrovesicular steatosis or lobular inflammation after a 24-week dietary challenge with fructose (18% calories) and fat (43% calories) in female Ossabaw pigs (starting from 6 months of age)^12^. However, when this diet was later supplemented with 2% w/w cholesterol, juvenile female Ossabaw pigs developed severe NASH with macrovesicular steatosis and lobular inflammation after 16 weeks^13^. Also, recently, Schumacher‑Petersen et al. described a Göttingen minipig model without significant steatosis but with significantly elevated inflammation and fibrosis after feeding a diet with ~ 18% fructose and 1–2% cholesterol over 13 months^14^.

These reports do not indicate a clear role for fructose in inducing NALFD/NASH histological changes in pigs; yet very high fructose-enriched diets similar to those applied in rodents have not been tested. Our aim in the present study was to evaluate if short-term feeding with very high fructose can increase hepatic DNL leading to steatosis and NAFLD in castrated male Danish Landrace-Yorkshire-Duroc pigs.

## Results

### Animal characteristics

Groups showed identical weight gain throughout the study period weighing 71.3 ± 1.9 kg and 72.0 ± 2.6 kg at euthanasia for standard chow (SC) and high-fructose (HF) pigs, respectively. Similarly, back fat thickness was similar measuring 2.6 ± 0.3 cm for SC and 2.6 ± 0.7 cm for HF.

### Biochemical characteristics

**Figure 1:**
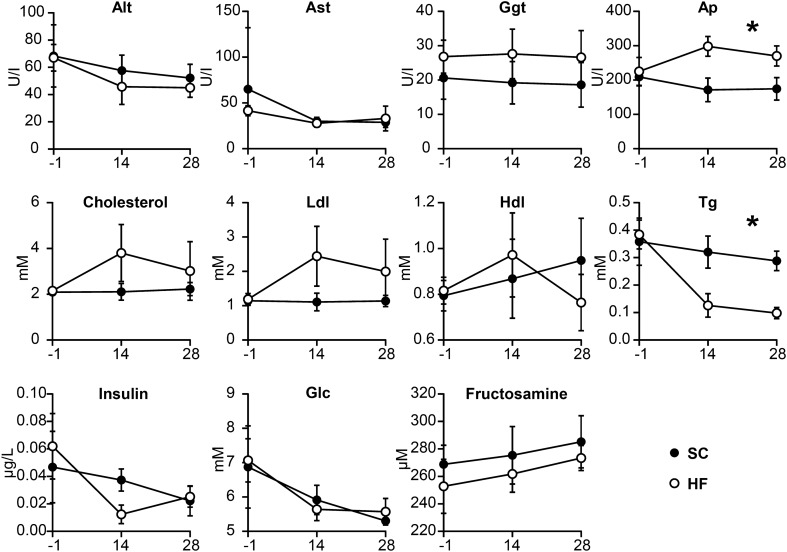
Biochemical characteristics between standard chow (SC) and high-fructose (HF) pigs. HF pigs had higher alkaline phosphatase (Ap) and lower triglyceride (Tg) levels during the study. For other abbreviations see main test. X-axis depicts days relative to study start. Values are mean ± SD. Groups were compared by AUC and significant differences are indicated *p < 0.05, Student’s t-test.

Blood sample analyses are depicted graphically in Fig. 1. HF pigs had significantly less plasma triglycerides (Tg) over the study period (5.14 ± 1.00 mM‧days compared with SC-pigs 9.00 ± 1.51 mM‧days, p = 0.02, Student’s t-test), and plasma alkaline phosphatase (Ap) activity was significantly higher (7640 ± 786 U/I‧days compared with SC pigs 5085 ± 776 U/I‧days, *p* = 0.02, Student’s t-test) as measured by the area under the curve (AUC). Since aspartate aminotransferase (Ast), alanine aminotransferase (Alt), and gamma-glutamyltransferase (Ggt) were all similar between the two groups, it is less likely that liver injury was the cause of the increased Ap which could stem from other tissues. Other measured parameters showed no significant differences between groups.

### Histological findings

**Figure 2:**
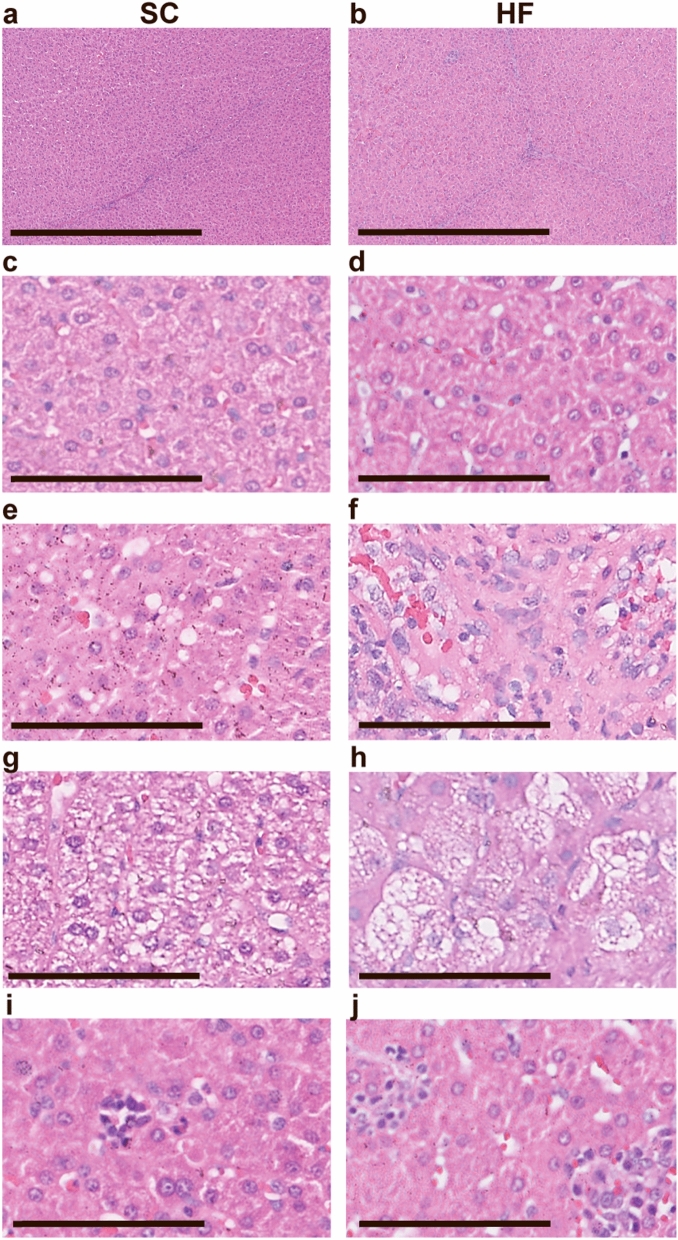
Representative liver HE stainings of pigs on standard chow (left panel, SC) and high-fructose (right panel, HF). Liver histology overall appeared normal (**a**–**d**) in both groups. Small foci with signs of microvesicular steatosis (**e**, **f**), hepatocyte ballooning (**g**, **h**) and inflammatory islets (**i**, **j**) could be found. Scalebars 800 µm (**a**, **b**) and 100 µm (**c**–**j**).

**Figure 3:**
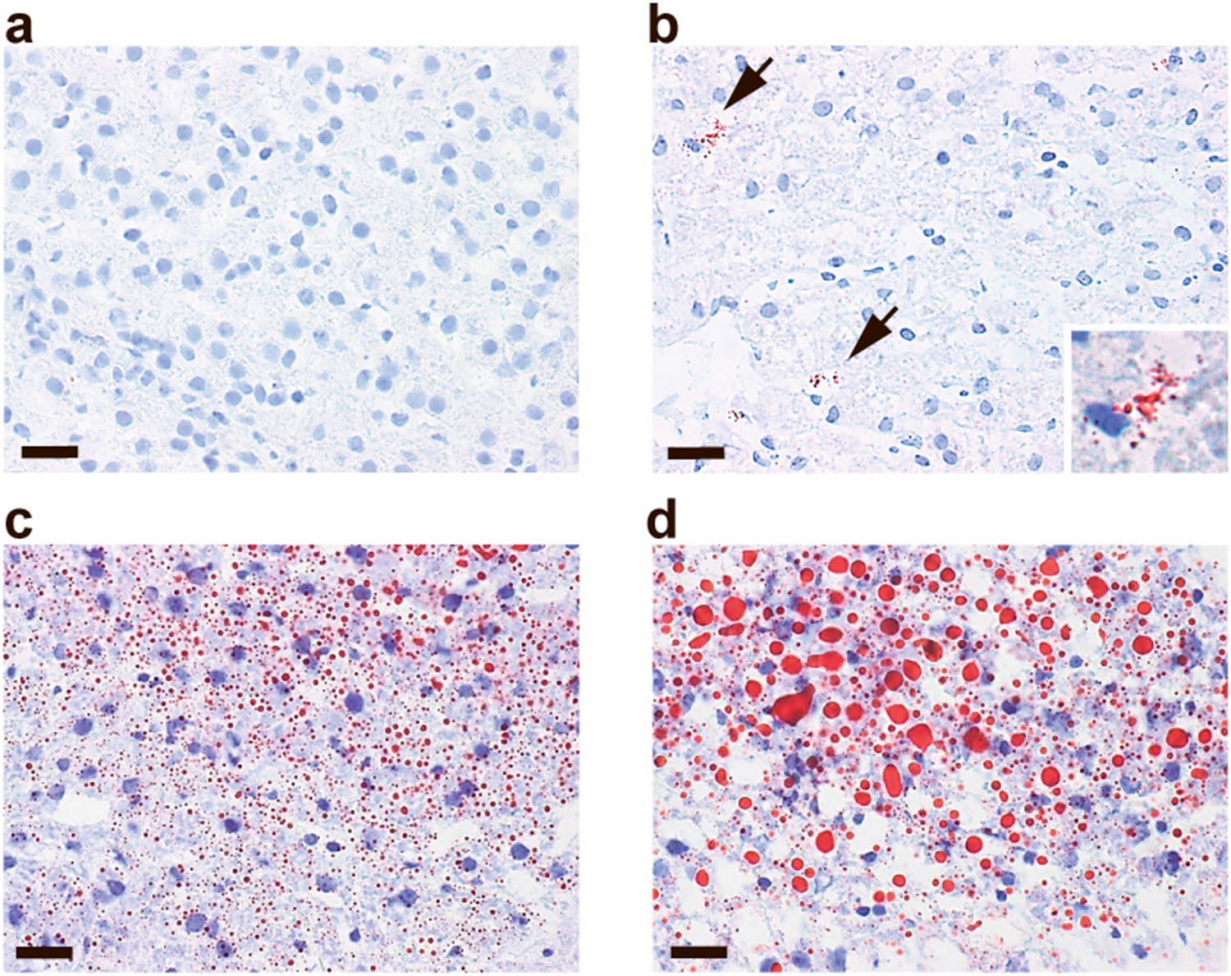
Hepatic lipid accumulation visualized by Oil Red O staining of frozen liver sections from pigs on SC (**a**) and HF (**b**). Arrow heads indicate areas of Oil Red O staining in HF pigs (upper area is magnified in lower right corner). Oil Red O positive control stainings from rats with fructose-induced steatosis were included to demonstrate micro- (**c**) and macrovesicular fatty droplets (**d**). Scalebars 20 µm.

**Figure 4:**
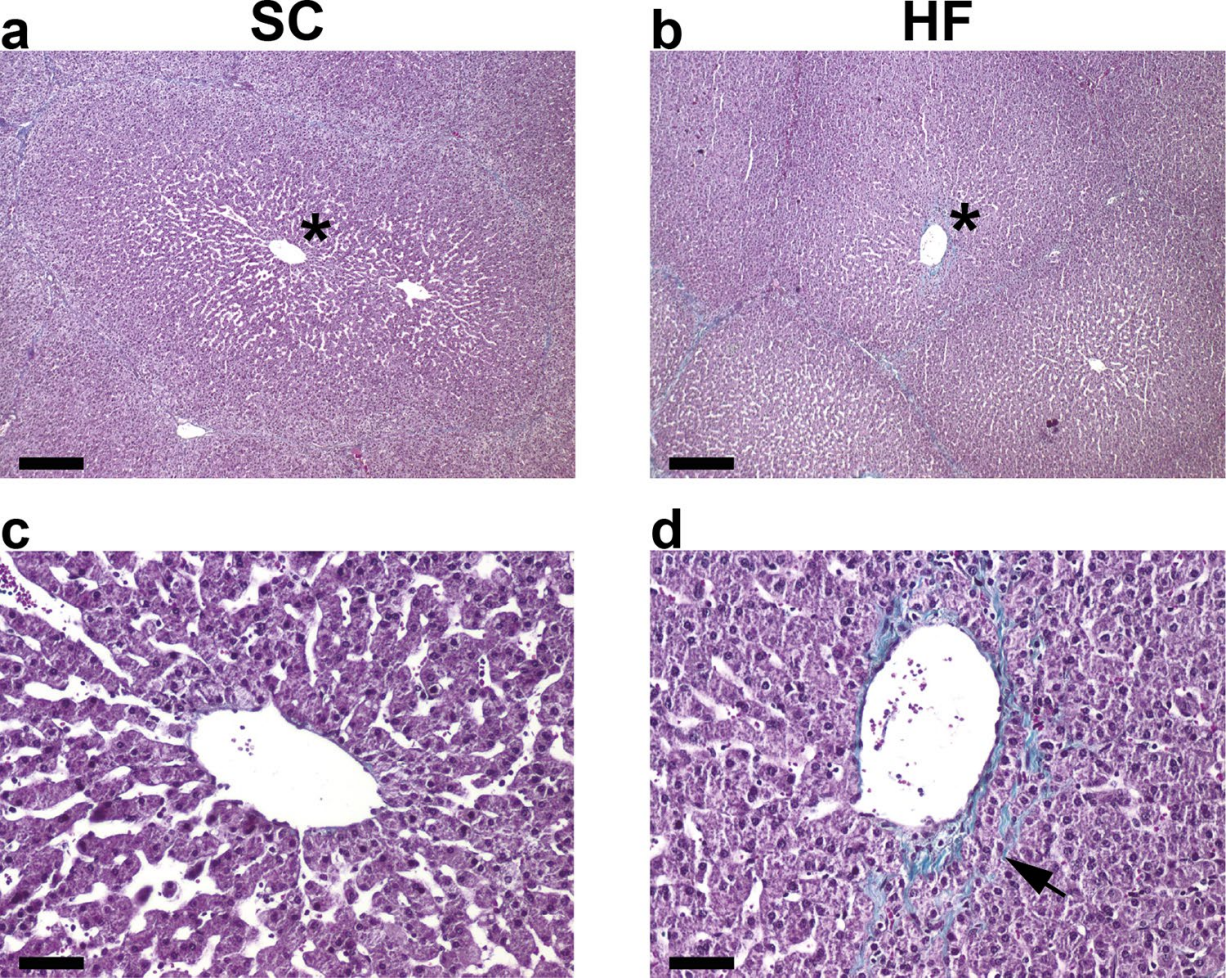
Representative liver Masson’s Trichrome staining of interlobular connective tissue in SC (**a**) and HF (**b**) pigs. An example of grade 1 perisinusoidal zone 3 fibrosis is depicted in a HF pig (arrow head, **d**) although grade 0 was more frequently observed in HF pigs. A normal central vein graded 0 is depicted for a SC pig (**c**). Areas in (**a**) and (**b**) (*) are magnified in c and d, respectively. Scalebars 200 µm (**a**, **b**) and 40 µm (**c**, **d**).

Minor areas of microvesicular steatosis and inflammatory foci as well as moderate hepatocyte ballooning were present in both groups (Fig. 2). Bright-field microscopic evaluation of haematoxylin-and-eosin (HE) stained sections did not reveal significant differences between the SC and HF groups with respect to steatosis or hepatocyte ballooning (Table 1), and steatosis did not in any case exceed the 5% threshold used to define the presence of human NAFLD^15^. HF pigs exhibited a minor, but statistically significant, increase in the presence of inflammatory islands compared with SC pigs, but overall NAFLD Activity Score (NAS) was not significantly different. Oil Red O staining, which allows sensitive detection of intracellular Tg and cholesterol esters, showed very limited lipid accumulation/deposition in the livers of HF pigs and no Oil Red O staining was detected in the SC pigs (Fig. 3). Masson’s Trichrome staining of interlobular and perisinusoidal zone 3 (near the central vein) connective tissue showed a small, but significant, elevated amount in HF compared with SC pigs, but severe fibrosis was not observed in either group (Table 1). Representative images are shown in Fig. 4.

**Table 1:** Histopathological scoring of liver tissue according to Kleiner et al.^15^.

Treatment group	SC (n = 5)	HF (n = 5)
Steatosis (0–3)	0.05 ± 0.04	0.02 ± 0.02
Inflammation (0–3)	0.05 ± 0.04*	0.12 ± 0.05*
Hepatocyte ballooning (0–2)	0.2 ± 0.08	0.11 ± 0.06
NAS	0.30 ± 0.13	0.25 ± 0.13
Fibrosis (0–4)	0.11 ± 0.05*	0.19 ± 0.02*

The NAFLD Activity Score (NAS) is the sum of the steatosis, lobular inflammation, and hepatocyte ballooning scores. Values are mean ± SD. Significant differences between groups are indicated by *p < 0.05, Student’s t-test.

### Markers of hepatic and adipose de novo* lipogenesis*

To investigate metabolic mechanisms related to fructose-driven DNL, we performed RNA profiling of key activators and enzymes involved in fatty acid metabolism (Table 2). Expression of DNL-related genes were found to be substantially higher in adipose than liver tissue, and the relative gene expression in adipose tissue of DNL key enzymes and regulators was significantly increased by fructose feeding, including ACLY, ACACA, FASN, INSIG1, and THRSP.

**Table 2:** Liver and adipose RNA profiling.

	Diet group	HF liver vs SC liver	HF adipose vs SC adipose	SC adipose vs SC liver	HF adipose vs HF liver
*Gene symbol*	*Encoded protein*				
**Fatty acid metabolism**					
ACACA	Acetyl-CoA carboxylase α	1.7↓**	2.0↑ **	6.6↑****	16↑****
ACADL	Acyl-CoA dehydrogenase. long chain	NS	NS	3.6↑****	3.8↑****
ACLY	ATP citrate lyase	1.8↓**	3.5↑ **	32↑****	143↑****
CPT1A	Carnitine palmitoyltransferase 1A	NS	1.7↓*	3.3↓****	NS
ELOVL6	Elongation of very long chain fatty acids 6	NS	1.5↑ *	15↑****	34↑****
FASN	Fatty acid synthase	2.8↓****	1.6↑*	21↑****	109↑****
INSIG1	Insulin induced gene 1	2.4↓***	1.7↑*	2.8↓***	NS
LEP	Leptin	NS	NS	86↑****	114↑****
ME1	Malic Enzyme 1	NS	NS	159↑****	NS
PKR	Translation initiation factor 2 alpha kinase 2	NS	NS	1.9↑***	NS
PPARG	Peroxisome proliferator activated receptor γ	NS	NS	NS	107↑****
SCD	Stearoyl-CoA desaturase	NS	1.9↑*	94↑****	163↑****
SREBF1	Sterol binding transcription factor 1	1.9↑*	NS	4.7↑****	3↑****
THRSP	Thyroid hormone responsive protein	2.5↓*	1.9↑*	20↑****	92↑****
**Macrophage biomarker**					
CD209	CD209 Molecule (M2)	NS	2.9↓*	3.3↑**	NS
CD163	CD163 Molecule (M2)	1.4↑*	NS	NS	2,9↓****
CD68	CD68 Molecule (M1/M2)	NS	NS	2.6↓***	NS
CD86	CD86 Molecule (M1)	1.7↑*	NS	NS	NS

The columns show significant fold change in regulation of gene expression between the indicated groups. Arrows indicate up-(↑) or down (↓) regulation in the first compared with the second mentioned group. Each data point represents two tissue samples from each pig and two replicates per sample. Groups were compared pair-wise using Student’s t-test or the Mann Whitney Wilcoxon Test, and significant differences are indicated by *p < 0.05, **p < 0.01, ***p < 0.001, and ****p < 0.0001. NS; not significant different.

**Figure 5:**
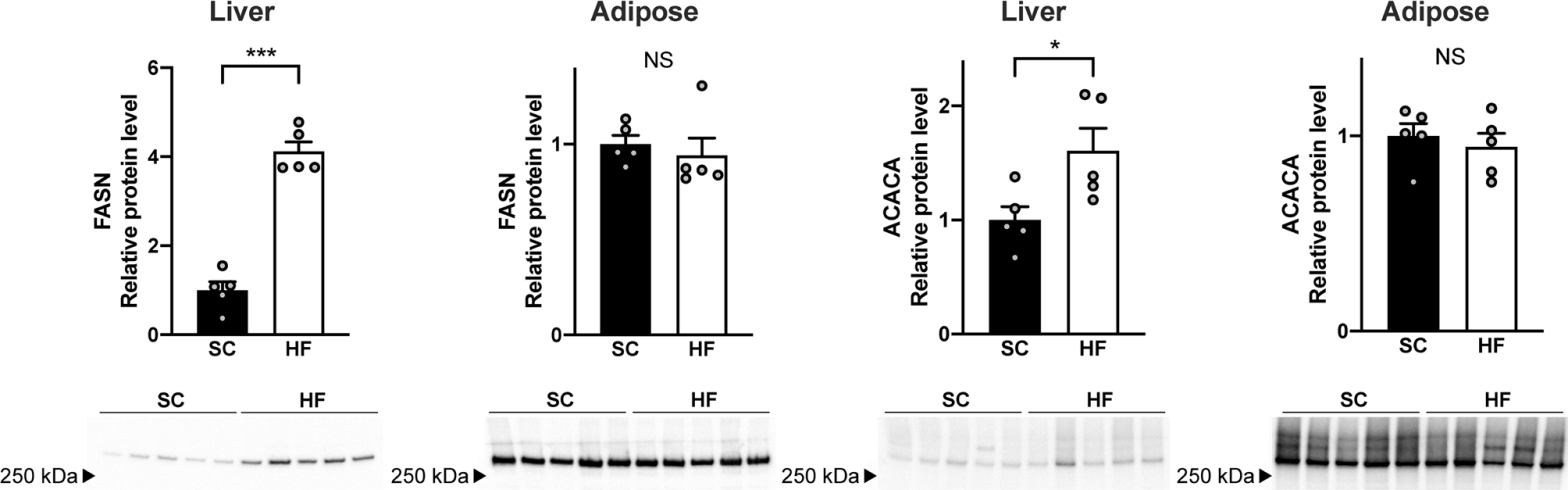
FASN and ACACA protein levels. High-fructose (HF) feeding leads to upregulation of FASN and ACACA protein in liver. Protein levels in adipose tissue were not significantly different between standard chow (SC) and HF groups. Protein levels were normalized to total protein measured in gels before transfer. Separate gels were used for “Liver FASN”, “Adipose FASN”, “Liver ACACA”, and “Adipose ACACA”. Uncut gels can be found in Supplementary information S1, Fig. 1. Significant differences between groups are indicated by *p < 0.05 and ***p < 0.001 (Student’s t-tests).

In liver, gene expression of the lipogenic sterol regulatory element binding transcription factor 1 (SREBF1) was up-regulated in HF compared with SC pigs, while expression of the lipogenic activators insulin induced gene 1 (INSIG1) and thyroid hormone-inducible hepatic protein (THRSP) as well as the key DNL enzymes acetyl-CoA carboxylase alpha (ACACA), ATP citrate lyase (ACLY), and fatty acid synthase (FASN) were all surprisingly down-regulated. ACACA and FASN undergo large diurnal variations linked to feeding/fasting. This makes mRNA levels difficult to interpret in pigs fed different diets that may be digested and metabolized at different rates. We therefore analysed ACACA and FASN on the protein level, where diurnal changes are less pronounced^16^. Here we found significant upregulation of hepatic ACACA and FASN in HF pigs, while levels in adipose tissues were unchanged. ACACA and FASN protein levels (5 ug total protein) were substantially lower in liver compared with adipose tissue (Fig. 5). The RNA profiling also included a panel of cytokine and cell specific markers (Supplementary information S1, Table 1). No significant changes in cytokines were noted, but there was a moderate up-regulation of some of the M1- and M2-macrophage markers in the livers of fructose feed pigs, consistent with the histological findings.

## Discussion

Fructose-induced hepatic DNL is undoubtably a contributing mechanism in human NAFLD, and very high levels of dietary fructose is an established tool to induce histopathological changes of NAFLD in rodents^3,7–10^. Previous efforts to establish porcine NAFLD models for translational research have also included fructose feeding, although the link between the dietary level of fructose and the histopathological outcome in pigs has not been clear. Species-specific differences in the balance between hepatic and adipose tissue DNL could be involved, but it is also possible that the moderate levels of dietary fructose in previous pig studies were not sufficient to induce the hepatic changes seen in rodents.

In the present study, we therefore evaluated the hepatic effects of feeding a very high fructose diet (60% w/w) for 4 weeks in castrated male Danish Landrace-Yorkshire-Duroc pigs. Pigs tolerated the diet well, however, and quite surprisingly, the diet did not cause any significant macrovesicular steatosis or hepatocyte ballooning.

Our results cast light over the role of fructose in previous diets used to induce NAFLD in pigs. Liang et al. previously showed that Ossabaw pigs fed a fructose-enriched (18% of energy) and high-fat (43% of energy) diet developed more than twofold increases in the plasma hepatic injury markers Ast and Alt after 8 weeks of feeding^12^. This was followed by hepatocyte ballooning and fibrosis, but not lobular inflammation or macrovesicular steatosis, after 24 weeks. The complete lack of changes in plasma Alt, Ast, and Ggt in the present study, where we fed a much higher level of fructose, indicates that the liver injury in the study of Liang et al. was likely not caused by the fructose content of the specialized diet. Importantly, the diet in that study also had reduced choline, i.e. 700 ppm; much less than the diet in the present study, which contained ~ 1765 ppm choline. Choline is an essential nutrient used to, among other things, synthesize phosphatidylcholine, which are major parts of mammalian cell membranes and important for secretion of very-low density lipoproteins that export lipids from hepatocytes. Dietary deficiency of choline has previously been shown to exacerbate steatosis in rodents, although the relevance of the disease mechanism for human NAFLD is unclear^17–19^. Consistently, another study in Ossabaw swine in which pigs were fed 6000 kcal/day of a moderate fructose-enriched diet (20% of energy) with a higher level of choline (1200 ppm) did not demonstrate Ast and Alt elevations or histological NASH or steatosis after 24 weeks of feeding^11^. This may indicate that choline-deficiency and feeding with a high fat content, rather than the moderate level of fructose alone, produced the histopathological and biochemical changes of NALFD previously reported in pigs. This interpretation is supported by a recent study that compared a moderate fructose-enriched diet with choline/methionine-deficient diets and found that only the latter induced steatosis in Göttingen minipigs^20^.

Fructose consumption increases hepatic DNL in humans, which in turn has been implicated in development of steatosis^21,22^. However, to understand the lack of fructose-induced steatosis in pigs, we therefore profiled key DNL enzymes in hepatic and adipose tissue. RNA profiling showed downregulation rather than the expected induction in HF livers, while expression was increased in adipose tissue. It is well known that hepatic DNL gene expression undergo large variations with feeding and fasting^16^. Consequently, the surprising reduction in hepatic mRNA levels could potentially be caused by rapid metabolism of the HF diet resulting in a longer period of fasting before euthanasia. Indeed, on the protein level (where circadian variations are less pronounced^16^), the key DNL enzymes FASN and ACACA were higher in HF pigs. These changes are consistent with fructose-induced regulation of hepatic DNL in rodents and humans. Fructose-induced steatohepatitis in rats is associated with significant hepatic up-regulation of DNL proteins, including FASN and ACACA^7,23,24^. In humans, several-fold increments of hepatic ACACA and FASN have also been described in steatotic livers, and blockade with a liver-specific allosteric inhibitor of ACACA significantly reduced human hepatic DNL after a fructose challenge^25,26^.

What sets pigs apart from rodents and human, however, is that adipose tissue is the major tissue responsible for DNL in pigs, while rodents and humans rely on hepatocytes for DNL^27–29^. Although HF feeding upregulated hepatic DNL enzymes, our study confirm that pigs utilize adipose tissue as the main DNL organ based on substantially higher ACACA and FASN protein levels (per total protein) compared to hepatic protein levels. This may explain why fructose-induced DNL in the pigs of the present study were not of a sufficient magnitude to cause hepatic steatosis. A feature also described in Göttingen minipigs where a diet moderate in fructose and cholesterol mediated inflammation and fibrosis but no significant steatosis after 13 months^14^. It is possible that this metabolic characteristic also underlies the relative resistance of another pig strain to develop other features of the metabolic syndrome, e.g. high plasma triglycerides, when fed fructose-enriched diets^30^.

Although the fructose-enriched diet did not cause steatosis, we did see minor increases in connective tissue and inflammatory islands on liver histology as well as upregulation of macrophage-expressed genes in liver homogenates. The culprit for these changes may have been the high content of cholesterol (2% w/w) that we introduced in the fructose-enriched diet to facilitate the potential development of NAFLD and NASH. Numerous previous studies have implicated cholesterol accumulation as a mediator for development of hepatic inflammation and fibrosis^31,32^. It is also possibly that gut dysbiosis caused by the fructose-enriched diet is implicated leading to increased gut permeability and endotoxin-driven hepatic inflammation as previously described in pigs and mice^13,33^.

Limitations to this study was that we cannot exclude that some steatosis could develop after longer feeding periods. However, our aim was to test whether short-term feeding with a high-fructose enriched diet induces hepatic steatosis and NAFLD similar to rodent models where such changes are seen already after 2–5 weeks^7–9^. The duration of the study was sufficient to observe minor increases in connective tissue and inflammatory cells, hence the diet did affect the liver metabolism, but no signs of steatosis was observed. As discussed previously, other mechanisms may be involved in pig hepatic steatosis and inflammation seen in other studies, e.g. gut dysbiosis and hepatic cholesterol accumulation, however, our study was not designed to assess these aspects, hence, other models with more robust inflammation would be more relevant to explore such mechanisms.

In conclusion, short-term feeding with high-fructose diet, which efficiently induce NAFLD in rodents, does not cause hepatic steatosis or NAFLD in pigs.

## Methods

### Animals and experimental design

Castrated male Danish Landrace-Yorkshire-Duroc pigs, bought from a SPF farm facility, were included. All time points are indicated relative to study start, where the pigs weighed 48.83 ± 1.90 kg (mean ± SD) and were approximately 3 months of age. From − 2 weeks they were kept at a 12/12 h day/night cycle in single booths with free access to water and sawdust on the floor in a large stable with snout contact to other pigs. From − 1 week they were presented with 6500 kcal/day SC to adapt to an increased feeding load. At study start, five pigs were randomized to receive a HF diet containing 60% fructose, 10% corn starch, 18% fat derived from soybean oil, 2% cholesterol, and 10% protein (Research Diets, Inc., New Brunswick, NJ, USA), while the other five pigs continued on SC, which contained 7% monosaccharides, 74.6% corn and wooden starch, 3.4% fat derived from palm oil and natural occurring fatty acids in wheat, rye, barley, oat, soybean and sunflower meal, and 15% protein (DLG, Copenhagen, Denmark). All pigs were fed twice a day totalling 6500 kcal/day, and any residual feed was removed and weighted at the end of each day. In both groups, the diet was completely consumed during the entire period except for small amounts not eaten by three HF pigs (representing 0.001%, 0.002%, and 0.009% of the feed offered). The experiment was approved by The Animal Experiments Inspectorate, Danish Veterinary and Food Administration, Ministry of Environment and Food of Denmark (Study approval license: 2015-15-0201-00570) and conducted in accordance with the Danish Ministry of Environment and Food animal research act.

### Blood and tissue samples

Baseline blood samples were drawn at − 1 week from the jugular vein after an overnight fast in lithium-heparin- and fluoride-citrate-EDTA-coated tubes, and plasma was stored at – 80 °C until later analyses. Blood sampling was repeated at 2 weeks and again at 4 weeks (the latter prior to euthanasia).

Euthanasia was managed by a captive bolt applied to the forehead with subsequent exsanguination. The liver was quickly removed and 4 tissue blocks, representing different parts of the liver, were preserved in 4% formaldehyde for 24 h and hereafter kept in phosphate-buffered saline at 4 °C until histological processing. Two tissue blocks from different random liver locations were placed in OCT and snap-frozen in liquid nitrogen and afterwards stored at − 80 °C. Tissue samples from 2 liver and back fat locations were immediately snap-frozen on dry ice and stored at − 80 °C until RNA purification and gene expression analysis. Subsequently, back fat thickness was measured along the posterior spine and the maximum distance from the skin to the osseous spine reported.

### Histology

Formaldehyde-fixed tissues were paraffin-embedded, sectioned (3 µm) and subsequent stained with HE to evaluate steatosis, inflammation and hepatocyte ballooning, and Masson’s Trichrome to evaluate fibrosis. Steatosis was further evaluated by Oil-Red-O staining of 4 µm sections cut from snap-frozen OCT-embedded tissue blocks. Slides were scanned with a NanoZoomer comprising four liver sections from each pig. Two observers (NHS and PS) randomly and independently divided each slide into 10 frames at × 20 magnification and graded the NAS (sum of steatosis 0–3; lobular inflammation 0–3; ballooning 0–2; values ≥ 5 was regarded as NASH) and the degree of fibrosis (0–4) as described by Kleiner et al^15^. Average scores were calculated for each pig for statistical analysis. All observations were made blinded to the study group and major inter-observatory differences resolved by consensus agreement.

### Biochemistry

Blood plasma glucose, Tg, Ast, Alt, Ggt, Ap, total cholesterol, low-density lipoprotein- (Ldl) and high-density lipoprotein- (Hdl) cholesterol, was determined according to standard procedures (Siemens Diagnostics Clinical Methods). Plasma fructosamine was determined by a spectrophotometric assay (reduction of nitrotetrazolium-blue, Roche Diagnostics GmbH, D-68298 Mannheim). All analyses were performed using an autoanalyzer, ADVIA 1800 Chemistry System (Siemens Medical Solutions, Tarrytown, NY 10,591, USA). Measurements of plasma insulin (porcine specific) were conducted by a commercial ELISA assay (Mercodia Porcine Insulin; Uppsala, Sweden). The instructions given by the manufacturer was followed.

### RNA profiling

Total RNA from liver and fat was purified using the RNeasy Mini Kit and the RNeasy Lipid Mini Kit, respectively (Qiagen, København Ø, Denmark). RT^2^ Profiler™ PCR Arrays with de novo lipogenesis and cytokine biomarkers were designed for Fluidigm® BioMark™ analysis (see Supplementary information S1, Table 1, for target and primer details). Reverse-transcription of RNA and preamplification of genes of interest was done using the Fluidigm Reverse Transcription Master Mix and the Fluidigm PreAmp Master Mix (Fluidigm Corporation) in accordance with the manufacturer’s instructions. In short, for initial cDNA synthesis, 0.1 ng of RNA was mixed with the RT mastermix (5x) in a 1:1 ratio and diluted 5 times in RNAse-free H_2_O followed by incubation at the following conditions: 25 °C for 5 min, 42 °C for 30 min and finally 85 °C for 5 min. To increase sensitivity, cDNAwas pre-amplified by pooling the Fluidigm DELTAgene Assays (Fluidigm Corporation) allowing a final concentration of 500 nM per assay. In the final pre-amplification reaction mixture, cDNA was diluted 1:4 with Fluidigm PreAmp Master Mix (5X), pooled deltagene assays (10X) and H_2_O. Reaction mixture was incubated at the following PCR conditions: 95 °C for 2 min followed by 14 cycles of 95 °C for 15 s and 60 °C for 4 min. To remove unincorporated primers, pre-amplified cDNA was incubated with 1,25U/µl Exonuclease I at 37 °C for 30 min followed by 80 °C for 15 min. After Exonuclease I treatment, pre-amplified cDNA was diluted 1:5 with TE buffer (10 mM Tris–HCl, 1 mM disodium EDTA, pH 8.0) and stored at − 20 °C. Gene expression analysis was carried out using the 48.48 dynamic arrays and Biomark HD system from Fluidigm (Fluidigm Corporation). Specifically, a 6 μl sample mixture was prepared for each sample containing 1 × SsoFast EvaGreen Supermix with low ROX (BioRad), 1 × DNA Binding Dye Sample Loading Reagent and each of diluted pre-amplified samples. Six μl of Assay mix was prepared with 1 × Assay Loading Reagent, 100 µM of each of the different Deltagene Assays and TE buffer. The 48.48 dynamic arrays were primed in an IFC controller before addition of samples and assay mixes in the appropriate inlets and loading in the IFC controller. After loading, the chip was placed in the BioMark HD Instrument for an initial thermal mix that included incubation at 70 °C for 40 min followed by 60 °C for 0.5 min. Subsequent qPCR was performed by 1 cycle of hot start at 95 °C for 1 min, followed by 30 cycles at 96° C for 5 s and 60 °C for 20 s with fluorescent recording after each cycle. For analysis of PCR products, the qPCR run was followed by a melting curve analysis where the fluorescent signal from dsDNA was measured at 60–95°C with 1 °C increment. The data was analyzed with Real-Time PCR Analysis Software (Fluidigm Corporation.). GenEx version 6.1 was used to analyze CT values and to calculate changes in gene expression. The relative expression levels of each target gene was normalized to beta-2-microglobulin, glyceraldehyde-3-phosphate dehydrogenase and hypoxanthine phosphoribosyltransferase (NormFinder) and calculated by the 2-delta delta Ct method^34^. The complete panel of analysed markers are shown in Supplementary information S1, Table 1.

### Western blotting

Total protein was extracted from liver and fat using RIPA buffer supplemented with protease inhibitor cocktail (cOmplete from Millipore-Sigma) with a tissue homogenizer (2X 30 s at 6000 rpm). Then samples were incubated with gentle agitation for 30 min at 4 degrees, followed by centrifugation for 20 min at 13 000 rpm. 5 ug protein lysates were run on 4–15% Criterion TGX Precast Gel (Bio-Rad, Denmark). Proteins were transferred to a nitrocellulose membrane (Hybond ECL, GE Healthcare, UK). After blocking (5% BSA at room temperature for one hour), the membranes were incubated overnight at 4 °C with the following primary antibodies: Anti-Acetyl-Coenzyme Carboxylase antibody (EP687Y) (ab45174), and Anti-Fatty Acid Synthase antibody (ab22759), both 1:4000 dilution from Abcam. The membranes were incubated with goat anti-rabbit horseradish peroxidase (HRP)-conjugated secondary antibodies for 1 h at room temperature (Dako, P0448 diluted 1:4000) and afterward, the antigen–antibody complex was visualized using an enhanced chemiluminescence system (ECL, Amersham ECL Plus, GE Healthcare). All Western blots were normalized to total protein, measured using Stain-Free technology, a method previously validated by others^35,36^. The total protein gel image can be found in Supplementary information S1, Fig. 2.

### Statistical analysis

All data variables were tested for normal distribution. Normally distributed variables are presented as mean ± SD. For comparison of the two groups, normally distributed data were tested with Student’s t-test and non-normally distributed data with the Mann Whitney Wilcoxon Test. Group differences in plasma analytes were assessed by comparing AUC (Welch’s-test was used for parameters with unequal variances) using GraphPad prism version 6.0 for Windows. RNA profiling data were analyzed by comparison of two groups as described above. *p*-values < 0.05 were considered significant.

## Supplementary Information

Supplementary Information.

## Data Availability

Data and protocols can be obtained by contacting the corresponding author.
