# Supplementary material for: High-fructose feeding does not induce steatosis or non-alcoholic fatty liver disease in pigs
Source: Sci Rep. 2021 Feb 2;11:2807. doi: 10.1038/s41598-021-82208-1 (PMC7854584; doi:10.1038/s41598-021-82208-1)
Supplement: Supplementary file 1 — Supplementary Information. [file 41598_2021_82208_MOESM1_ESM.pdf]

Supplementary information S1

Title: High-fructose feeding does not induce steatosis or non-alcoholic fatty liver disease in pigs

Authors: Nikolaj H. Schmidt, Pia Svendsen, Julián Albarrán-Juárez, Søren K. Moestrup, and Jacob Fog Bentzon

Figure 1: Uncut gels – Western blot

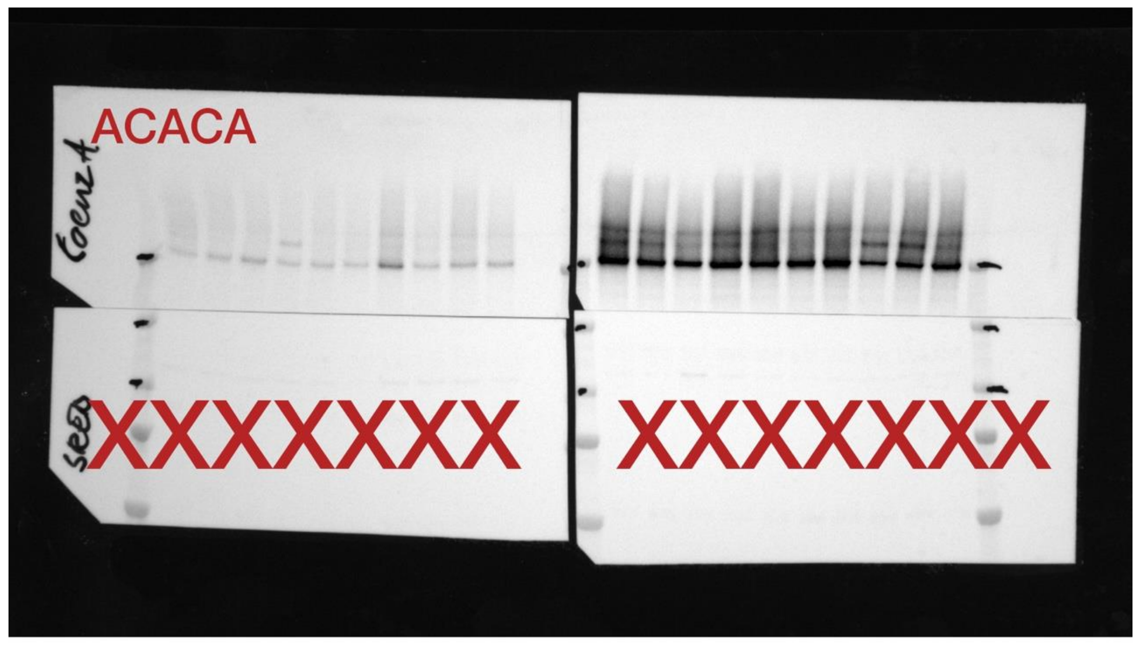

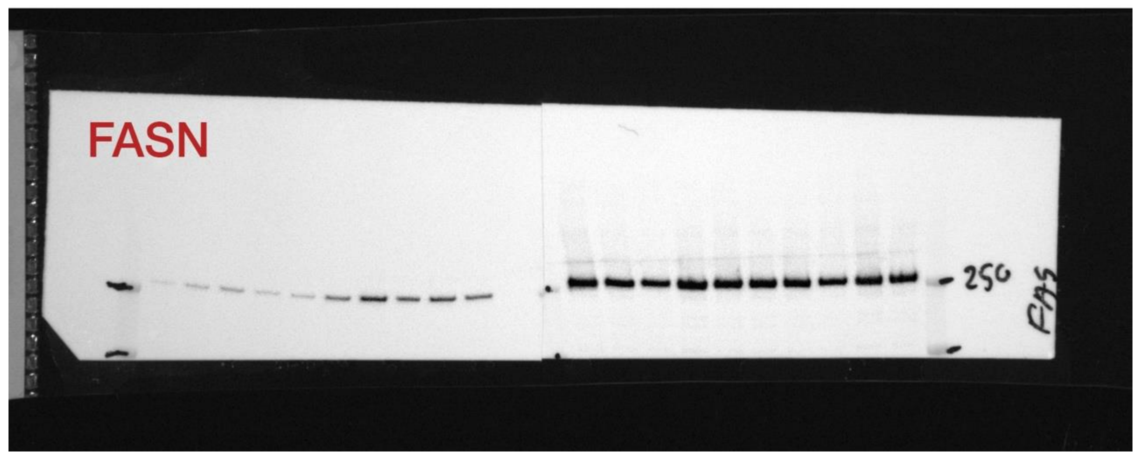

Uncut gels. Only the high molecular part of the membrane was used to stain for ACACA and FASN. Part of a low molecular part of the membrane is also shown in the figure but this part was not stained with ACACA or FASN.

Figure 2: Stain-free image of total protein loading that were used for normalization – Western blot

FASN

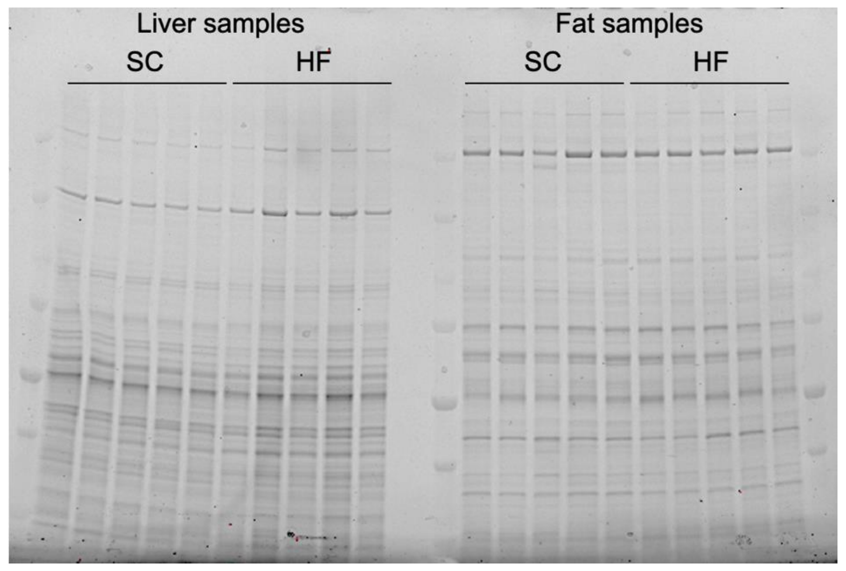

Criterion TGX Stain-Free 4-15% gel.

ACACA

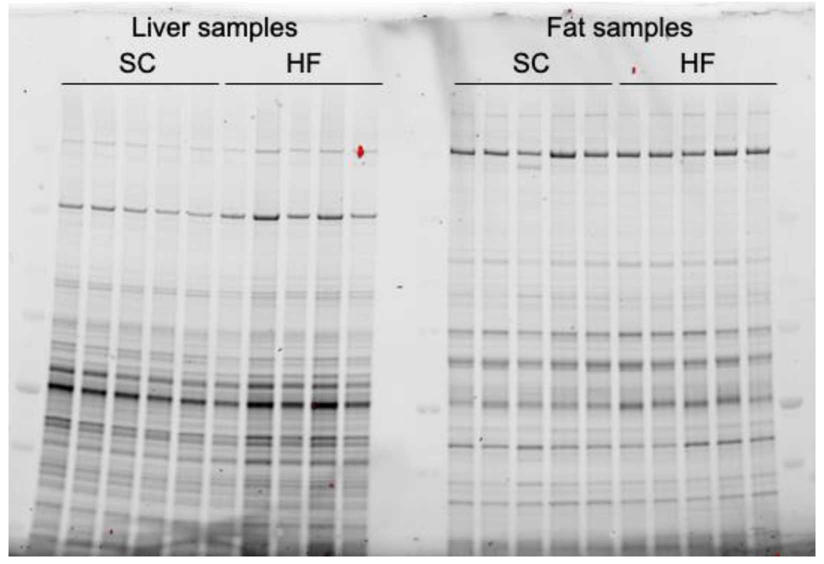

Criterion TGX Stain-Free 4-15% gel.

Table 1: RNA-profiling – target and primer details

<table>
<tr><th>Target</th><th>Assay ID</th><th>Assay Name</th><th>FP</th><th>RP</th><th>Design RefSeq</th><th>Blast Hits</th><th>Gene Symbol</th><th>Gene Aliases</th><th>Gene Full Name</th><th>GO Function</th><th>GO Process</th><th>GO Component</th></tr>
<tr><td>ACACA</td><td>GEP00084830</td><td>ACACA_84830_i39</td><td>ATCCCAGCTGATCCTGCAAA</td><td>GCAGAGTCTGGAAACCAAACC</td><td>NM_001114269.N</td><td>NM_001114269</td><td>ACACA</td><td></td><td>acetyl-CoA carboxylase alpha</td><td></td><td></td><td></td></tr>
<tr><td>ACACA</td><td>GEP00084941</td><td>ACACA_84941_i29</td><td>CAGCCTGTACAAGGAAGTGAC</td><td>ATGGTCCCTGTTTGTCTCCA</td><td>NM_001114269.N</td><td>NM_001114269</td><td>ACACA</td><td></td><td>acetyl-CoA carboxylase alpha</td><td></td><td></td><td></td></tr>
<tr><td>ACADL</td><td>GEP00084831</td><td>ACADL_84831_i5</td><td>GTTAAGGGGCGAAAGCTACA</td><td>CTGGCAACCGTACATCTTCA</td><td>NM_213897.N</td><td>NM_213897</td><td>ACADL</td><td></td><td>acyl-CoA dehydrogenase, long chain</td><td>flavin adenine dinucleotide binding|long-chain-acyl-CoA dehydrogenase activity</td><td>long-chain fatty acid catabolic process|oxidation-reduction process</td><td>mitochondrial matrix</td></tr>
<tr><td>ACADL</td><td>GEP00084949</td><td>ACADL_84949_i8</td><td>TCCAGCTGCATGAAACGAAAC</td><td>GCCACGCTGTTTTGTAACTCA</td><td>NM_213897.N</td><td>NM_213897</td><td>ACADL</td><td></td><td>acyl-CoA dehydrogenase, long chain</td><td>flavin adenine dinucleotide binding|long-chain-acyl-CoA dehydrogenase activity</td><td>long-chain fatty acid catabolic process|oxidation-reduction process</td><td>mitochondrial matrix</td></tr>
<tr><td>ACLY</td><td>GEP00084939</td><td>ACLY_84939_i3</td><td>CCAGAGGCTTCCTCAAGAAC</td><td>GCAGCGTAGATGCAGACATA</td><td>NM_001257276.N</td><td>NM_001257276|NM_001105302</td><td>ACLY</td><td>ACL</td><td>ATP citrate lyase</td><td></td><td></td><td></td></tr>
<tr><td>ACLY</td><td>GEP00084940</td><td>ACLY_84940_i4</td><td>GCTGAATCCTGAGGCCATCAA</td><td>CGGAGATAAAGCTGGCCAGAA</td><td>NM_001257276.N</td><td>NM_001257276|NM_001105302</td><td>ACLY</td><td>ACL</td><td>ATP citrate lyase</td><td></td><td></td><td></td></tr>
<tr><td>B2M</td><td>GEP00084818</td><td>B2M_84818_i2</td><td>CGAGACCACTAACCGGCATCA</td><td>TGGATTCATCCAACCCAGATGCA</td><td>NM_213978.N</td><td>NM_213978</td><td>B2M</td><td></td><td>beta-2-microglobulin</td><td></td><td>antigen processing and presentation of peptide antigen via MHC class I|immune response</td><td>MHC class I protein complex|extracellular region</td></tr>
<tr><td>CD163</td><td>GEP00084814</td><td>CD163_84814_e0</td><td>GTCTGCTCTCTGGCAGTGTA</td><td>CATGTCACACCAGCATCTTCA</td><td>NM_213976.N</td><td>NM_213976</td><td>CD163</td><td></td><td>CD163 molecule</td><td>scavenger receptor activity|virus receptor activity</td><td>acute-phase response|receptor-mediated endocytosis|viral entry into host cell</td><td>extracellular region|integral component of membrane|plasma membrane</td></tr>
<tr><td>CD209</td><td>GEP00084840</td><td>CD209_84840_i3</td><td>AGAGAGAGACCAGCAGGAGAC</td><td>CAGGTCATCTGCTGCAGGAA</td><td>NM_001129972.N</td><td>NM_001129972</td><td>CD209</td><td>DC-SIGN</td><td>CD209 molecule</td><td></td><td></td><td></td></tr>
<tr><td>CD28</td><td>GEP00084821</td><td>CD28_84821_i0</td><td>GGGTTACTCCTGGCTCTCAA</td><td>AGTATGGGCGACTGCTTCA</td><td>NM_001287410.N</td><td>NM_001287410</td><td>CD28</td><td>TP44</td><td>CD28 molecule</td><td></td><td></td><td></td></tr>
<tr><td>CD68</td><td>GEP00084847</td><td>CD68_84847_i3</td><td>ACATGGCTGTGGAGTACAAC</td><td>TGGAGATCTCGAAGGGATGAA</td><td>NM_001291776.N</td><td>NM_001291776</td><td>CD68</td><td></td><td>CD68 molecule</td><td></td><td></td><td></td></tr>
<tr><td>CD80</td><td>GEP00084817</td><td>CD80_84817_i0</td><td>AAGCGGGAGAGAGGGTCTTA</td><td>GGCAGTAATACTAGGCACAGGAA</td><td>NM_214087.N</td><td>NM_214087</td><td>CD80</td><td>B7-1</td><td>CD80 molecule</td><td></td><td></td><td></td></tr>
<tr><td>CD86</td><td>GEP00084846</td><td>CD86_84846_i4</td><td>ATGAACAGGAAGGCGAGTGAA</td><td>ACATCACACTGGGCATCATCA</td><td>NM_214222.N</td><td>NM_214222</td><td>CD86</td><td></td><td>CD86 molecule</td><td></td><td></td><td></td></tr>
<tr><td>CD8A</td><td>GEP00084844</td><td>CD8A_84844_i0</td><td>ATGGCCTCGCTGGTGAC</td><td>CAAGCTGGACCCGAGGAC</td><td>NM_001001907.N</td><td>NM_001001907</td><td>CD8A</td><td></td><td>CD8a molecule</td><td></td><td></td><td></td></tr>
<tr><td>CEBPB</td><td>GEP00084838</td><td>CEBPB_84838_e0</td><td>CGCAGGTCAAGAGTAAGACCAA</td><td>GCTCACGCCGGATCTTGTA</td><td>NM_001199889.N</td><td>NM_001199889</td><td>CEBPB</td><td></td><td>CCAAT/enhancer binding protein beta</td><td></td><td></td><td></td></tr>
<tr><td>CPT1A</td><td>GEP00084827</td><td>CPT1A_84827_i2</td><td>ACTGGACCGAGAGGAAATCA</td><td>GGAATCCGGGACGTGTTAAA</td><td>NM_001129805.N</td><td>NM_001129805</td><td>CPT1A</td><td>CPT1|L-CPTI</td><td>carnitine palmitoyltransferase 1A</td><td></td><td></td><td></td></tr>
<tr><td>CPT1A</td><td>GEP00084946</td><td>CPT1A_84946_i0</td><td>AAAGTCCTGGTGGGCTACAA</td><td>TCGTCCTCGGAGGTAGATGTA</td><td>NM_001129805.N</td><td>NM_001129805</td><td>CPT1A</td><td>CPT1|L-CPTI</td><td>carnitine palmitoyltransferase 1A</td><td></td><td></td><td></td></tr>
<tr><td>CPT1B</td><td>GEP00084948</td><td>CPT1B_84948_i10</td><td>TTCACCCTCATCGCGTTCAA</td><td>CCCGCGATCATGTAGGAGAC</td><td>NM_001007191.N</td><td></td><td>CPT1B</td><td>CPT1|CPT1-M|M-CPTI</td><td>carnitine palmitoyltransferase 1B</td><td>carnitine O-palmitoyltransferase activity</td><td>fatty acid beta-oxidation|transport</td><td>integral component of membrane|mitochondrial outer membrane</td></tr>
<tr><td>CSF2</td><td>GEP00084835</td><td>CSF2_84835_i2</td><td>ACTCTGTTGGCCAAGCACTA</td><td>AAGGTGATAGACTGGGTTTCACA</td><td>NM_214118.N</td><td>NM_214118</td><td>CSF2</td><td>GM-CSF</td><td>colony stimulating factor 2</td><td>cytokine activity|granulocyte macrophage colony-stimulating factor receptor binding|growth factor activity</td><td>ERK1 and ERK2 cascade|TOR signaling|immune response|interferon-tau production|phosphatidylinositol 3-kinase signaling|positive regulation of trophectodermal cell proliferation|protein kinase B signaling</td><td>extracellular space|intracellular</td></tr>
<tr><td>PKR</td><td>GEP00084950</td><td>PKR_84950_i2</td><td>AGACAGGACCTGCACATAAC</td><td>CTACCTTCAGCTTTGGGGAA</td><td>NM_214319.N</td><td>NM_214319</td><td>EIF2AK2</td><td>PKR|PRKR</td><td>eukaryotic translation initiation factor 2 alpha kinase 2</td><td></td><td></td><td></td></tr>
<tr><td>FASN</td><td>GEP00084832</td><td>FASN_84832_i9</td><td>TGGAGGGGCTATTGCATCAA</td><td>TGCTTACACTCTTCCCAGGAC</td><td>NM_001099930.N</td><td>NM_001099930</td><td>FASN</td><td></td><td>fatty acid synthase</td><td></td><td></td><td></td></tr>
<tr><td>FASN</td><td>GEP00084942</td><td>FASN_84942_i13</td><td>GGGACAACCTGGAGTTCTTCC</td><td>AACAGACCGTTGGGGTTGAC</td><td>NM_001099930.N</td><td>NM_001099930</td><td>FASN</td><td></td><td>fatty acid synthase</td><td></td><td></td><td></td></tr>
<tr><td>RN18S_manual</td><td>GEP00084958</td><td>RN18S_manual_84958</td><td>GCCCTCGGTCGAGTTGTC</td><td>CTTGCAGGGCGGTGACAG</td><td>FLDM-028286.1</td><td></td><td>FLDM-028286</td><td></td><td></td><td></td><td></td><td></td></tr>
<tr><td>ELOVL6_manual</td><td>GEP00084956</td><td>ELOVL6_manual_84956</td><td>AGCAGTTCAACGAGAACGAAGCC</td><td>TGCCGACCGCCAAAGATAAAG</td><td>FLDM-032674.1</td><td></td><td>FLDM-032674</td><td></td><td></td><td></td><td></td><td></td></tr>
<tr><td>ME1(malic enzyme 1)</td><td>GEP00084804</td><td>FLDM-080917_84804_e0</td><td>ACTTGGCTTTCACCCTGGAA</td><td>CCTGGATGTCCTGACTGATGAA</td><td>FLDM-080917.1</td><td>FLDM-080917.1</td><td>FLDM-080917</td><td></td><td></td><td></td><td></td><td></td></tr>
<tr><td>GAPDH</td><td>GEP00084833</td><td>GAPDH_84833_i1</td><td>TGTCTTCACGACCATGGAGAA</td><td>GTTCACGCCCATCACAAACA</td><td>NM_001206359.N</td><td>NM_001206359</td><td>GAPDH</td><td>GAPD</td><td>glyceraldehyde-3-phosphate dehydrogenase</td><td>NAD binding|NADP binding|glyceraldehyde-3-phosphate dehydrogenase (NAD+) (phosphorylating) activity|microtubule binding|peptidyl-cysteine S-nitrosylase activity</td><td>glucose metabolic process|glycolytic process|microtubule cytoskeleton organization|neuron apoptotic process|oxidation-reduction process|peptidyl-cysteine S-trans-nitrosylation|protein stabilization|regulation of translation</td><td>GAIT complex|cytoplasm|cytosol|microtubule cytoskeleton|nucleus</td></tr>
<tr><td>GK</td><td>GEP00084815</td><td>GK_84815_i16</td><td>TCACAGTGGAGCGGTTTGAA</td><td>ATCACGGCTTTCTTCCACGTA</td><td>NM_001143708.N</td><td>NM_001143708</td><td>GK</td><td></td><td>glycerol kinase</td><td></td><td></td><td></td></tr>
<tr><td>GK</td><td>GEP00084944</td><td>GK_84944_i2</td><td>GTGAGAAACTTGGACAGCTCAA</td><td>ACAGTGGTTTCCCTCTGGTTA</td><td>NM_001143708.N</td><td>NM_001143708</td><td>GK</td><td></td><td>glycerol kinase</td><td></td><td></td><td></td></tr>
<tr><td>HPRT1</td><td>GEP00084822</td><td>HPRT1_84822_i5</td><td>CCCCTCGAAGTGTTGGCTATA</td><td>AAGGGCATAGCCTACCACAAA</td><td>NM_001032376.N</td><td>NM_001032376</td><td>HPRT1</td><td>HPRT</td><td>hypoxanthine phosphoribosyltransferase 1</td><td>guanine phosphoribosyltransferase activity|hypoxanthine phosphoribosyltransferase activity|magnesium ion binding|nucleotide binding|protein homodimerization activity</td><td>GMP catabolic process|IMP metabolic process|IMP salvage|guanine salvage|hypoxanthine metabolic process|hypoxanthine salvage|positive regulation of dopamine metabolic process|protein homotetramerization|purine nucleotide biosynthetic process|purine ribonucleoside salvage</td><td>cytoplasm</td></tr>
<tr><td>IFNG</td><td>GEP00084824</td><td>IFNG_84824_i2</td><td>TGGTAGCTCTGGGAAACTGAA</td><td>ATGGCTTTGCGCTGGATC</td><td>NM_213948.N</td><td>NM_213948</td><td>IFNG</td><td></td><td>interferon gamma</td><td>cytokine activity|interferon-gamma receptor binding</td><td>CD8-positive, alpha-beta T cell differentiation involved in immune response|T cell receptor signaling pathway|adaptive immune response|antigen processing and presentation|cell cycle arrest|cellular response to interleukin-18|cellular response to lipopolysaccharide|defense response to bacterium|defense response to protozoan|defense response to virus|endoplasmic reticulum unfolded protein response|extrinsic apoptotic signaling pathway|humoral immune response|interferon-gamma-mediated signaling pathway|negative regulation of growth of symbiont in host|negative regulation of interleukin-17 production|negative regulation of myelination|negative regulation of smooth muscle cell proliferation|negative regulation of transcription, DNA-templated|neutrophil apoptotic process|neutrophil chemotaxis|positive regulation of CD4-positive, CD25-positive, alpha-beta regulatory T cell differentiation involved in immune response|positive regulation of MHC class II biosynthetic process|positive regulation of T cell proliferation|positive regulation of autophagy|positive regulation of calcidiol 1-monooxygenase activity|positive regulation of chemokine biosynthetic process|positive regulation of core promoter binding|positive regulation of epithelial cell migration|positive regulation of exosomal secretion|positive regulation of fructose 1,6-bisphosphate 1-phosphatase activity|positive regulation of fructose 1,6-bisphosphate metabolic process|positive regulation of interleukin-1 beta secretion|positive regulation of interleukin-12 biosynthetic process|positive regulation of interleukin-23 production|positive regulation of interleukin-6 biosynthetic process|positive regulation of isotype switching to IgG isotypes|positive regulation of killing of cells of other organism|positive regulation of membrane protein ectodomain proteolysis|positive regulation of nitric oxide biosynthetic process|positive regulation of osteoclast differentiation|positive regulation of peptidyl-serine phosphorylation of STAT protein|positive regulation of protein complex assembly|positive regulation of protein deacetylation|positive regulation of protein import into nucleus, translocation|positive regulation of protein serine/threonine kinase activity|positive regulation of smooth muscle cell apoptotic process|positive regulation of transcription from RNA polymerase II promoter|positive regulation of tumor necrosis factor (ligand) superfamily member 11 production|positive regulation of tumor necrosis factor production|positive regulation of tyrosine phosphorylation of STAT protein|protein import into nucleus, translocation|regulation of defense response to virus by host|regulation of insulin secretion|regulation of protein ADP-ribosylation</td><td>external side of plasma membrane|extracellular space|intracellular</td></tr>
<tr><td>IL10</td><td>GEP00084841</td><td>IL10_84841_i2</td><td>GGAGAAGCTGAAGACCCTCA</td><td>CGGCCTTGCTCTTGTTTTCA</td><td>NM_214041.N</td><td>NM_214041</td><td>IL10</td><td>CSIF|IL-10</td><td>interleukin 10</td><td>cytokine activity</td><td>branching involved in labyrinthine layer morphogenesis|cellular response to hepatocyte growth factor stimulus|cellular response to lipopolysaccharide|defense response to bacterium|defense response to protozoan|immune response|inflammatory response|negative regulation of B cell proliferation|negative regulation of chronic inflammatory response to antigenic stimulus|negative regulation of cytokine activity|negative regulation of cytokine secretion involved in immune response|negative regulation of endothelial cell apoptotic process|negative regulation of growth of symbiont in host|negative regulation of heterotypic cell-cell adhesion|negative regulation of interferon-gamma production|negative regulation of interleukin-12 production|negative regulation of interleukin-6 production|negative regulation of membrane protein ectodomain proteolysis|negative regulation of myeloid dendritic cell</td><td>extracellular space</td></tr>
</table>

<table>
<tr><td></td><td></td><td></td><td></td><td></td><td></td><td></td><td></td><td></td><td></td><td></td><td>activation|negative regulation of tumor necrosis factor production|negative regulation of vascular smooth muscle cell proliferation|positive regulation of B cell apoptotic process|positive regulation of MHC class II biosynthetic process|positive regulation of cell cycle|positive regulation of cytokine secretion|positive regulation of endothelial cell proliferation|positive regulation of sequence-specific DNA binding transcription factor activity|positive regulation of transcription from RNA polymerase II promoter|receptor biosynthetic process|regulation of response to wounding|response to glucocorticoid</td><td></td></tr>
<tr><td>IL12A</td><td>GEP00084843</td><td>IL12A_84843_i6</td><td>TCTGGATCAGAACATGCTGACA</td><td>TGGCACAGTCTCACTGTTGAA</td><td>NM_213993.N</td><td>NM_213993</td><td>IL12A</td><td></td><td>interleukin 12A</td><td>cytokine activity|growth factor activity|interleukin-12 beta subunit binding|interleukin-12 receptor binding|interleukin-27 binding|protein heterodimerization activity</td><td>cell cycle arrest|cell migration|cellular response to lipopolysaccharide|cellular response to virus|defense response to Gram-positive bacterium|defense response to protozoan|extrinsic apoptotic signaling pathway|immune response|negative regulation of interleukin-17 production|negative regulation of smooth muscle cell proliferation|positive regulation of NK T cell activation|positive regulation of T cell differentiation|positive regulation of T cell mediated cytotoxicity|positive regulation of T cell proliferation|positive regulation of dendritic cell chemotaxis|positive regulation of interferon-gamma production|positive regulation of natural killer cell activation|positive regulation of natural killer cell mediated cytotoxicity directed against tumor cell target|positive regulation of smooth muscle cell apoptotic process|positive regulation of tyrosine phosphorylation of STAT protein|response to UV-B</td><td>cytoplasm|interleukin-12 complex</td></tr>
<tr><td>IL13</td><td>GEP00084845</td><td>IL13_84845_i1</td><td>CATGGTGTGGAGCGTCAAC</td><td>GGAGATGTTGATGAGGGATTCCA</td><td>NM_213803.N</td><td>NM_213803</td><td>IL13</td><td>L13|IL-13</td><td>interleukin 13</td><td>cytokine activity|cytokine receptor binding</td><td>cellular response to cytokine stimulus|immune response|inflammatory response|negative regulation of complement-dependent cytotoxicity|negative regulation of endothelial cell apoptotic process|positive regulation of immunoglobulin production|positive regulation of macrophage activation|positive regulation of mast cell degranulation</td><td>cytoplasm|external side of plasma membrane|extracellular space</td></tr>
<tr><td>IL1B1</td><td>GEP00084951</td><td>IL1B1_84951_i4</td><td>TGGCCCACACATGCTGAA</td><td>CCTTGCACAAAGCTCATGCA</td><td>NM_214055.N</td><td>NM_214055</td><td>IL1B</td><td>IL1B1</td><td>interleukin 1 beta</td><td>cytokine activity|interleukin-1 receptor binding</td><td>cytokine-mediated signaling pathway|fever generation|immune response|positive regulation of cell division|positive regulation of estradiol secretion</td><td>autophagosome|cytoplasmic vesicle|cytosol|extracellular exosome|extracellular space|lysosome</td></tr>
<tr><td>IL4</td><td>GEP00084839</td><td>IL4_84839_i2</td><td>TGAGCGGACTTGACAGGAA</td><td>GCTCTTCTTGGCTTCATGCA</td><td>NM_214123.N</td><td>NM_214123</td><td>IL4</td><td></td><td>interleukin 4</td><td>cytokine activity|growth factor activity|interleukin-4 receptor binding</td><td>B cell activation|B cell costimulation|T-helper 1 cell lineage commitment|T-helper 2 cell cytokine production|T-helper 2 cell differentiation|cholesterol metabolic process|defense response to protozoan|extrinsic apoptotic signaling pathway in absence of ligand|innate immune response in mucosa|myeloid dendritic cell differentiation|negative regulation of T-helper 17 cell differentiation|negative regulation of complement-dependent cytotoxicity|negative regulation of endothelial cell apoptotic process|negative regulation of epithelial cell migration|negative regulation of extrinsic apoptotic signaling pathway|negative regulation of osteoclast differentiation|negative regulation of transcription, DNA-templated|negative regulation of white fat cell proliferation|positive regulation of B cell proliferation|positive regulation of JAK-STAT cascade|positive regulation of MHC class II biosynthetic process|positive regulation of T cell differentiation|positive regulation of activated T cell proliferation|positive regulation of chemokine biosynthetic process|positive regulation of establishment of endothelial barrier|positive regulation of gene expression|positive regulation of interleukin-13 production|positive regulation of isotype switching to IgE isotypes|positive regulation of isotype switching to IgG isotypes|positive regulation of mast cell degranulation|positive regulation of myoblast fusion|positive regulation of protein phosphorylation|positive regulation of sequence-specific DNA binding transcription factor activity|positive regulation of transcription from RNA polymerase II promoter|positive regulation of tyrosine phosphorylation of STAT protein|regulation of protein export from nucleus</td><td>external side of plasma membrane|extracellular space</td></tr>
<tr><td>IL5</td><td>GEP00084819</td><td>IL5_84819_i1</td><td>ACTTGATGATTTCAACTCCTGTAC</td><td>TCAACGTGTCTATTCCCTGAA</td><td>NM_214205.N</td><td>NM_214205</td><td>IL5</td><td>IL-5</td><td>interleukin 5</td><td>cytokine activity|growth factor activity|interleukin-5 receptor binding</td><td>immune response|positive regulation of immunoglobulin secretion|positive regulation of peptidyl-tyrosine phosphorylation|positive regulation of podosome assembly|positive regulation of sequence-specific DNA binding transcription factor activity|positive regulation of transcription, DNA-templated</td><td>extracellular space</td></tr>
<tr><td>IL6</td><td>GEP00084823</td><td>IL6_84823_i0</td><td>AGGAACCCAGCTATGAACTCC</td><td>AGTAGCCATCACCAGAAGCA</td><td>NM_214399.N</td><td>NM_214399</td><td>IL6</td><td></td><td>interleukin 6</td><td>cytokine activity|growth factor activity|interleukin-6 receptor binding</td><td>T-helper 17 cell lineage commitment|acute-phase response|positive regulation of estradiol secretion</td><td>extracellular space</td></tr>
<tr><td>INSIG1</td><td>GEP00084955</td><td>INSIG1_84955_i2</td><td>CGGTTTTCGTTGGCATCAAC</td><td>AGCGTCAAGGAGAGCTGAA</td><td>NM_001244521.N</td><td>NM_001244521</td><td>INSIG1</td><td>CL-6</td><td>insulin induced gene 1</td><td></td><td></td><td></td></tr>
<tr><td>INSIG1</td><td>GEP00084957</td><td>INSIG1_84957_i1</td><td>ATTGCCACCATCTTCTCCTCA</td><td>CGATGCAGGGGTAGAGCAA</td><td>NM_001244521.N</td><td>NM_001244521</td><td>INSIG1</td><td>CL-6</td><td>insulin induced gene 1</td><td></td><td></td><td></td></tr>
<tr><td>LEP</td><td>GEP00084825</td><td>LEP_84825_i0</td><td>AGGATGACACCAAAACCCTCA</td><td>CCCTCTGTTTGGAGGAGACA</td><td>NM_213840.N</td><td>NM_213840</td><td>LEP</td><td>OB|OBS</td><td>leptin</td><td>growth factor activity|hormone activity|peptide hormone receptor binding</td><td>T cell differentiation|activation of protein kinase C activity|activation of protein kinase C activity|adipose tissue development|adult feeding behavior|angiogenesis|bile acid metabolic process|bone growth|cellular response to leptin stimulus|central nervous system neuron development|cholesterol metabolic process|eating behavior|energy reserve metabolic process|fatty acid beta-oxidation|glucose homeostasis|glucose metabolic process|hormone metabolic process|insulin secretion|interleukin-12 production|interleukin-6 production|interleukin-6 secretion|interleukin-8 secretion|intestinal absorption|leptin-mediated signaling pathway|lipid metabolic process|negative regulation of appetite by leptin-mediated signaling pathway|negative regulation of appetite by leptin-mediated signaling pathway|negative regulation of autophagy|negative regulation of glucagon secretion|negative regulation of glucose import|negative regulation of transcription from RNA polymerase II promoter|phagocytosis|placenta development|positive regulation of JAK-STAT cascade|positive regulation of MAPK cascade|positive regulation of STAT protein import into nucleus|positive regulation of T cell proliferation|positive regulation of TOR signaling|positive regulation of TOR signaling|positive regulation of developmental growth|positive regulation of myeloid cell differentiation|positive regulation of p38MAPK cascade|positive regulation of p38MAPK cascade|positive regulation of phosphatidylinositol 3-kinase signaling|positive regulation of phosphatidylinositol 3-kinase signaling|positive regulation of protein kinase B signaling|positive regulation of tumor necrosis factor production|prostaglandin secretion|regulation of angiogenesis|regulation of bone remodeling|regulation of brown fat cell differentiation|regulation of cell cycle|regulation of cytokine production involved in inflammatory response|regulation of endothelial cell proliferation|regulation of gluconeogenesis|regulation of insulin secretion|regulation of intestinal cholesterol absorption|regulation of natural killer cell activation|regulation of natural killer cell mediated cytotoxicity|regulation of natural killer cell proliferation|regulation of nitric-oxide synthase activity|regulation of steroid biosynthetic process|response to dietary excess|response to insulin|response to insulin|sexual reproduction|tyrosine phosphorylation of STAT protein</td><td>cytoplasm|extracellular space</td></tr>
<tr><td>LEP</td><td>GEP00084945</td><td>LEP_84945_i0</td><td>GTCCAGGATGACACCAAAACC</td><td>CCCTCTGTTTGGAGGAGACA</td><td>NM_213840.N</td><td>NM_213840</td><td>LEP</td><td>OB|OBS</td><td>leptin</td><td>growth factor activity|hormone activity|peptide hormone receptor binding</td><td>T cell differentiation|activation of protein kinase C activity|activation of protein kinase C activity|adipose tissue development|adult feeding behavior|angiogenesis|bile acid metabolic process|bone growth|cellular response to leptin stimulus|central nervous system neuron development|cholesterol metabolic process|eating behavior|energy reserve metabolic process|fatty acid beta-oxidation|glucose homeostasis|glucose metabolic process|hormone metabolic process|insulin secretion|interleukin-12 production|interleukin-6 production|interleukin-6 secretion|interleukin-8 secretion|intestinal absorption|leptin-mediated signaling pathway|lipid metabolic process|negative regulation of appetite by leptin-mediated signaling pathway|negative regulation of appetite by leptin-mediated signaling pathway|negative regulation of autophagy|negative regulation of glucagon secretion|negative regulation of glucose import|negative regulation of transcription from RNA polymerase II promoter|phagocytosis|placenta development|positive regulation of JAK-STAT cascade|positive regulation of MAPK cascade|positive regulation of STAT protein import into nucleus|positive regulation of T cell proliferation|positive regulation of TOR signaling|positive regulation of TOR signaling|positive regulation of developmental growth|positive regulation of myeloid cell differentiation|positive regulation of p38MAPK cascade|positive regulation of p38MAPK cascade|positive regulation of phosphatidylinositol 3-kinase signaling|positive regulation of phosphatidylinositol 3-kinase signaling|positive regulation of protein kinase B signaling|positive regulation of tumor necrosis factor production|prostaglandin</td><td>cytoplasm|extracellular space</td></tr>
</table>

											secretion|regulation of angiogenesis|regulation of bone remodeling|regulation of brown fat cell differentiation|regulation of cell cycle|regulation of cytokine production involved in inflammatory response|regulation of endothelial cell proliferation|regulation of gluconeogenesis|regulation of insulin secretion|regulation of intestinal cholesterol absorption|regulation of natural killer cell activation|regulation of natural killer cell mediated cytotoxicity|regulation of natural killer cell proliferation|regulation of nitric-oxide synthase activity|regulation of steroid biosynthetic process|response to dietary excess|response to insulin|response to insulin|sexual reproduction|tyrosine phosphorylation of STAT protein	
PPARG	GEP00084834	PPARG_84834_i5	ACAGCGACCTGGCGATATTTA	TGGGCTTCACATTCAGCAAAC	NM_214379.N	NM_214379	PPARG		peroxisome proliferator activated receptor gamma	DBD domain binding|DNA binding|LBD domain binding|RNA polymerase II transcription factor activity, ligand-activated sequence-specific DNA binding|activating transcription factor binding|alpha-actinin binding|chromatin binding|core promoter sequence-specific DNA binding|drug binding|enzyme binding|identical protein binding|ligand-dependent nuclear receptor transcription coactivator activity|peptide binding|protein C-terminus binding|protein heterodimerization activity|protein self-association|retinoid X receptor binding|steroid hormone receptor activity|transcription factor activity, sequence-specific DNA binding|transcription regulatory region DNA binding|zinc ion binding	activation of cysteine-type endopeptidase activity involved in apoptotic process|cell maturation|cellular response to insulin stimulus|glucose homeostasis|lipoprotein transport|low-density lipoprotein particle receptor biosynthetic process|macrophage derived foam cell differentiation|monocyte differentiation|negative regulation of cholesterol storage|negative regulation of gene expression|negative regulation of interferon-gamma-mediated signaling pathway|negative regulation of macrophage derived foam cell differentiation|negative regulation of receptor biosynthetic process|negative regulation of sequestering of triglyceride|negative regulation of smooth muscle cell proliferation|negative regulation of transcription from RNA polymerase II promoter|peroxisome proliferator activated receptor signaling pathway|positive regulation of DNA binding|positive regulation of cholesterol efflux|positive regulation of fat cell differentiation|positive regulation of intracellular cholesterol transport|positive regulation of prostaglandin biosynthetic process|positive regulation of protein secretion|positive regulation of sequence-specific DNA binding transcription factor activity|positive regulation of transcription from RNA polymerase II promoter|positive regulation of transcription, DNA-templated|positive regulation of transcription, DNA-templated|regulation of blood pressure|regulation of circadian rhythm|regulation of protein localization to plasma membrane|regulation of transcription involved in cell fate commitment|response to low-density lipoprotein particle stimulus|response to retinoic acid|rhythmic process|steroid hormone mediated signaling pathway|transcription, DNA-templated|white fat cell differentiation	RNA polymerase II transcription factor complex|cytoplasm|nucleus
SCD	GEP00084836	SCD_84836_i3	CGAGAAGCTGGTGATGTTCC	GGCAGGATGAAGCACATCAA	NM_213781.N	NM_213781	SCD		stearoyl-CoA desaturase	iron ion binding|oxidoreductase activity|stearoyl-CoA 9-desaturase activity	monounsaturated fatty acid biosynthetic process|oxidation-reduction process|unsaturated fatty acid biosynthetic process	endoplasmic reticulum membrane|integral component of membrane
SREBF1	GEP00084931	SREBF1_84931_e0	CGGTGCCTGCCTTCTC	CTGCAGGATGGTCAGTGG	NM_214157.N	NM_214157	SREBF1	ADD1|SREBP1|SCREBP1|SREBF1c|SREBP-1C	sterol regulatory element binding transcription factor 1	DNA binding|protein dimerization activity	cholesterol metabolic process|lipid biosynthetic process|positive regulation of triglyceride biosynthetic process|regulation of transcription, DNA-templated|transcription, DNA-templated	ER to Golgi transport vesicle membrane|Golgi membrane|cytoplasm|endoplasmic reticulum membrane|integral component of membrane|nucleus
TGFB1	GEP00084816	TGFB1_84816_e0	CATCGACATGGAGCTGGTGAA	GAGCCGAAGCTTGGACAGAA	NM_214015.N	NM_214015	TGFB1	TGF-BETA-1	transforming growth factor beta 1	antigen binding|cytokine activity|growth factor activity|protein binding|type I transforming growth factor beta receptor binding|type II transforming growth factor beta receptor binding|type II transforming growth factor beta receptor binding|type III transforming growth factor beta receptor binding	ATP biosynthetic process|BMP signaling pathway|MAPK cascade|SMAD protein complex assembly|SMAD protein import into nucleus|SMAD protein signal transduction|cell cycle arrest|cell death|cell development|cell growth|cell migration|cell-cell junction organization|cellular response to organic cyclic compound|cellular response to transforming growth factor beta stimulus|chondrocyte differentiation|common-partner SMAD protein phosphorylation|epidermal growth factor receptor signaling pathway|epithelial to mesenchymal transition|evasion or tolerance of host defenses by virus|extracellular matrix assembly|extrinsic apoptotic signaling pathway|hematopoietic progenitor cell differentiation|hyaluronan catabolic process|inflammatory response|lipopolysaccharide-mediated signaling pathway|lymph node development|membrane protein intracellular domain proteolysis|mitotic cell cycle checkpoint|negative regulation of DNA replication|negative regulation of blood vessel endothelial cell migration|negative regulation of cell cycle|negative regulation of cell growth|negative regulation of cell proliferation|negative regulation of cell-cell adhesion|negative regulation of epithelial cell proliferation|negative regulation of fat cell differentiation|negative regulation of gene expression|negative regulation of hyaluronan biosynthetic process|negative regulation of macrophage cytokine production|negative regulation of mitotic cell cycle|negative regulation of myoblast differentiation|negative regulation of protein phosphorylation|negative regulation of skeletal muscle tissue development|negative regulation of transcription, DNA-templated|ossification involved in bone remodeling|pathway-restricted SMAD protein phosphorylation|phosphate-containing compound metabolic process|positive regulation of ERK1 and ERK2 cascade|positive regulation of MAP kinase activity|positive regulation of NAD+ ADP-ribosyltransferase activity|positive regulation of SMAD protein import into nucleus|positive regulation of blood vessel endothelial cell migration|positive regulation of bone mineralization|positive regulation of cell division|positive regulation of cell migration|positive regulation of cell proliferation|positive regulation of cellular protein metabolic process|positive regulation of chemotaxis|positive regulation of collagen biosynthetic process|positive regulation of epithelial to mesenchymal transition|positive regulation of fibroblast migration|positive regulation of gene expression|positive regulation of interleukin-17 production|positive regulation of isotype switching to IgA isotypes|positive regulation of pathway-restricted SMAD protein phosphorylation|positive regulation of pathway-restricted SMAD protein phosphorylation|positive regulation of peptidyl-serine phosphorylation|positive regulation of peptidyl-threonine phosphorylation|positive regulation of phosphatidylinositol 3-kinase activity|positive regulation of protein complex assembly|positive regulation of protein dephosphorylation|positive regulation of protein import into nucleus|positive regulation of protein kinase B signaling|positive regulation of protein phosphorylation|positive regulation of protein secretion|positive regulation of superoxide anion generation|positive regulation of transcription from RNA polymerase II promoter|positive regulation of transcription regulatory region DNA binding|positive regulation of transcription, DNA-templated|protein export from nucleus|protein import into nucleus, translocation|protein kinase B signaling|protein phosphorylation|receptor catabolic process|regulation of DNA binding|regulation of MAPK cascade|regulation of SMAD protein import into nucleus|regulation of apoptotic process|regulation of binding|regulation of protein import into nucleus|regulation of striated muscle tissue development|regulation of transforming growth factor beta receptor signaling pathway|response to cholesterol|response to estradiol|response to progesterone|response to wounding|salivary gland morphogenesis|transforming growth factor beta receptor signaling pathway|transforming growth factor beta receptor signaling pathway	blood microparticle|cell surface|cytoplasm|extracellular space|extracellular space|microvillus|nucleus|proteinaceous extracellular matrix
THRSP	GEP00084837	THRSP_84837_i0	AGGAGGTGACGAGGAAATACC	GCCTTCCGTCGTGTAGAGTA	NM_001244376.N	NM_001244376	THRSP	SPOT14	thyroid hormone responsive			
THRSP	GEP00084943	THRSP_84943_i0	CTGAGGAGGTGACGAGGAAA	GCCTTCCGTCGTGTAGAGTA	NM_001244376.N	NM_001244376	THRSP	SPOT14	thyroid hormone responsive			
TNF	GEP00084820	TNF_84820_i1	CTGGCCCCTTGAGCATCA	GGGCTTATCTGAGGTTTGAGAC	NM_214022.N	NM_214022	TNF	TNFa	tumor necrosis factor	cytokine activity|identical protein binding|protease binding|transcription regulatory region DNA binding|tumor necrosis factor receptor binding	JNK cascade|activation of MAPK activity|activation of MAPKKK activity|activation of cysteine-type endopeptidase activity involved in apoptotic process|cellular response to amino acid stimulus|cellular response to nicotine|chronic inflammatory response to antigenic stimulus|cortical actin cytoskeleton organization|defense response to Gram-positive bacterium|embryonic digestive tract development|endothelial cell apoptotic process|epithelial cell proliferation involved in salivary gland morphogenesis|extracellular matrix organization|extrinsic apoptotic signaling pathway via death domain receptors|glucose metabolic process|humoral immune response|intrinsic apoptotic signaling pathway in response to DNA damage|leukocyte tethering or rolling|lipopolysaccharide-mediated signaling pathway|necroptotic signaling pathway|negative regulation of alkaline phosphatase activity|negative regulation of bicellular tight junction assembly|negative regulation of branching involved in lung morphogenesis|negative regulation of cytokine secretion involved in immune response|negative regulation of endothelial cell proliferation|negative regulation of extrinsic apoptotic signaling pathway in absence of ligand|negative regulation of glucose import|negative regulation of growth of symbiont in host|negative regulation of interleukin-6 production|negative regulation of lipid catabolic process|negative regulation of mitotic cell cycle|negative regulation of myoblast	external side of plasma membrane|extracellular space|integral component of plasma membrane|membrane raft|phagocytic cup|recycling endosome

differentiation|negative regulation of myosin-light-chain-phosphatase activity|negative regulation of osteoblast differentiation|negative regulation of protein complex disassembly|negative regulation of transcription from RNA polymerase II promoter|negative regulation of viral genome replication|osteoclast differentiation|positive regulation of JUN kinase activity|positive regulation of NF-kappaB import into nucleus|positive regulation of NF-kappaB transcription factor activity|positive regulation of NFAT protein import into nucleus|positive regulation of NIK/NF-kappaB signaling|positive regulation of blood microparticle formation|positive regulation of calcidiol 1-monooxygenase activity|positive regulation of chemokine (C-X-C motif) ligand 2 production|positive regulation of chemokine biosynthetic process|positive regulation of chronic inflammatory response to antigenic stimulus|positive regulation of cytokine secretion|positive regulation of endothelial cell apoptotic process|positive regulation of estradiol secretion|positive regulation of fever generation|positive regulation of hair follicle development|positive regulation of heterotypic cell-cell adhesion|positive regulation of humoral immune response mediated by circulating immunoglobulin|positive regulation of interferon-gamma production|positive regulation of interleukin-6 production|positive regulation of interleukin-8 biosynthetic process|positive regulation of leukocyte adhesion to arterial endothelial cell|positive regulation of membrane protein ectodomain proteolysis|positive regulation of nitric oxide biosynthetic process|positive regulation of osteoclast differentiation|positive regulation of peptidyl-serine phosphorylation|positive regulation of phagocytosis|positive regulation of podosome assembly|positive regulation of protein complex disassembly|positive regulation of protein kinase B signaling|positive regulation of protein localization to cell surface|positive regulation of superoxide dismutase activity|positive regulation of transcription from RNA polymerase II promoter|positive regulation of translational initiation by iron|positive regulation of vascular smooth muscle cell proliferation|protein import into nucleus, translocation|protein kinase B signaling|receptor biosynthetic process|regulation of branching involved in salivary gland morphogenesis|regulation of establishment of endothelial barrier|regulation of immunoglobulin secretion|regulation of insulin secretion|response to glucocorticoid|response to virus|sequestering of triglyceride|tumor necrosis factor-mediated signaling pathway
